# Expression and purification of soluble and active human enterokinase light chain in *Escherichia coli*

**DOI:** 10.1016/j.btre.2021.e00626

**Published:** 2021-05-05

**Authors:** Young Su Kim, Hye-Jeong Lee, Sang-hyun Park, Yeu-chun Kim, Jungoh Ahn

**Affiliations:** a Department of Chemical and Biomolecular Engineering, KAIST, Daejeon 34141, Republic of Korea; b Biotechnology Process Engineering Center, KRIBB, Cheongju 28116, Republic of Korea; c Department of Bioprocess Engineering, KRIBB School of Biotechnology, Korea University of Science and Technology (UST), 217 Gajeong-ro, Yuseong-gu, Daejeon 34113, Republic of Korea

**Keywords:** D_4_K, (Aspartic Acid)_4_ Lysine, EK, enterokinase, bEKL, bovine enterokinase light chain, hEKL, human enterokinase light chain, IPTG, isopropyl β-D-1-thiogalactopyranoside, MBP, maltose-binding protein, TEV, tobacco etch virus, Human enterokinase light chain, *Escherichia coli*, Recombinant protein, Fusion technology, Self-cleavage

## Abstract

Human enterokinase light chain (hEK_L_) specifically cleaves the sequence (Asp)_4_-Lys↓X (D_4_K), making this a frequently used enzyme for site-specific cleavage of recombinant fusion proteins. However, hEK_L_ production from *Escherichia coli* is limited due to intramolecular disulphide bonds. Here, we present strategies to obtain soluble and active hEK_L_ from *E. coli* by expressing the hEK_L_ variant C112S fused with maltose-binding protein (MBP) through D_4_K and molecular chaperons including GroEL/ES. The fusion protein self-cleaved *in vivo,* thereby removing the MBP in the *E. coli* cells. Thus, the self-cleaved hEK_L_ variant was released into the culture medium. One-step purification using HisTrap™ chromatography purified the hEK_L_ variant exhibiting an enzymatic activity of 3.1 × 10^3^ U/mL (9.934 × 10^5^ U/mg). The approaches presented here greatly simplify the purification of hEK_L_ from *E. coli* without requiring refolding processes.

## Highlights

• Recombinant production of soluble, active enterokinase (EK) is challenging.
• Maltose binding protein-fusion improves EK solubility but reduces activity.
• GroEL/ES and Erv2/PDI induces correct refolding and improves EK activity.
• Replacing free cysteine with serine dramatically improves EK activity.

Recombinant fusion technology has been used to enhance the expression level and solubility of target proteins, and to facilitate their purification [1,2]. Proteases including Factor Xa, thrombin, tobacco etch virus (TEV) protease, and enterokinase (EK) are used for the site-specific cleavage of recombinant tags from fusion proteins [[3], [4], [5], [6]]. While Factor Xa, thrombin, and TEV protease cleave inside the recognition site, EK cleaves outside the site, thus it has a proteolytic activity regardless of the P1′ position sequence.

Human EK (hEK) (DDDDK↓, D_4_K↓) is produced by cells in the duodenum and intestinal brush-border [[7], [8], [9]]. EK activates trypsin by cleavage of trypsinogen [[10], [11], [12]]. hEK consists of an 86 kDa heavy chain and a 28 kDa light chain that are connected by a single disulphide bond. The heavy chain contains an intestinal brush-border membrane-binding motif. The light chain harbours the classical catalytic triad (chymotrypsin His57, Asp102, and Ser195) with four intramolecular disulphide bonds. The hEK light chain (hEK_L_) can cleave the fusion protein to obtain the authentic form of the protein [13]. In addition, hEK_L_ is an attractive protease for use in protein purification due to its broad range of reaction conditions (pH 4.5–9.5 and temperature 4–45 °C), tolerance against various detergents, and reusability [10,12].

hEK_L_ has a 10-fold higher catalytic efficiency (k_cat_/K_M_) than bEK_L_ [14,15]. However, several reports show that hEK_L_ is expressed in inclusion bodies in *E. coli* [10] that necessitates refolding using dialysis [[16], [17], [18], [19]], dilution [18,[20], [21], [22]], or on-column methods [18,[23], [24], [25]].

**Fig. 1:**
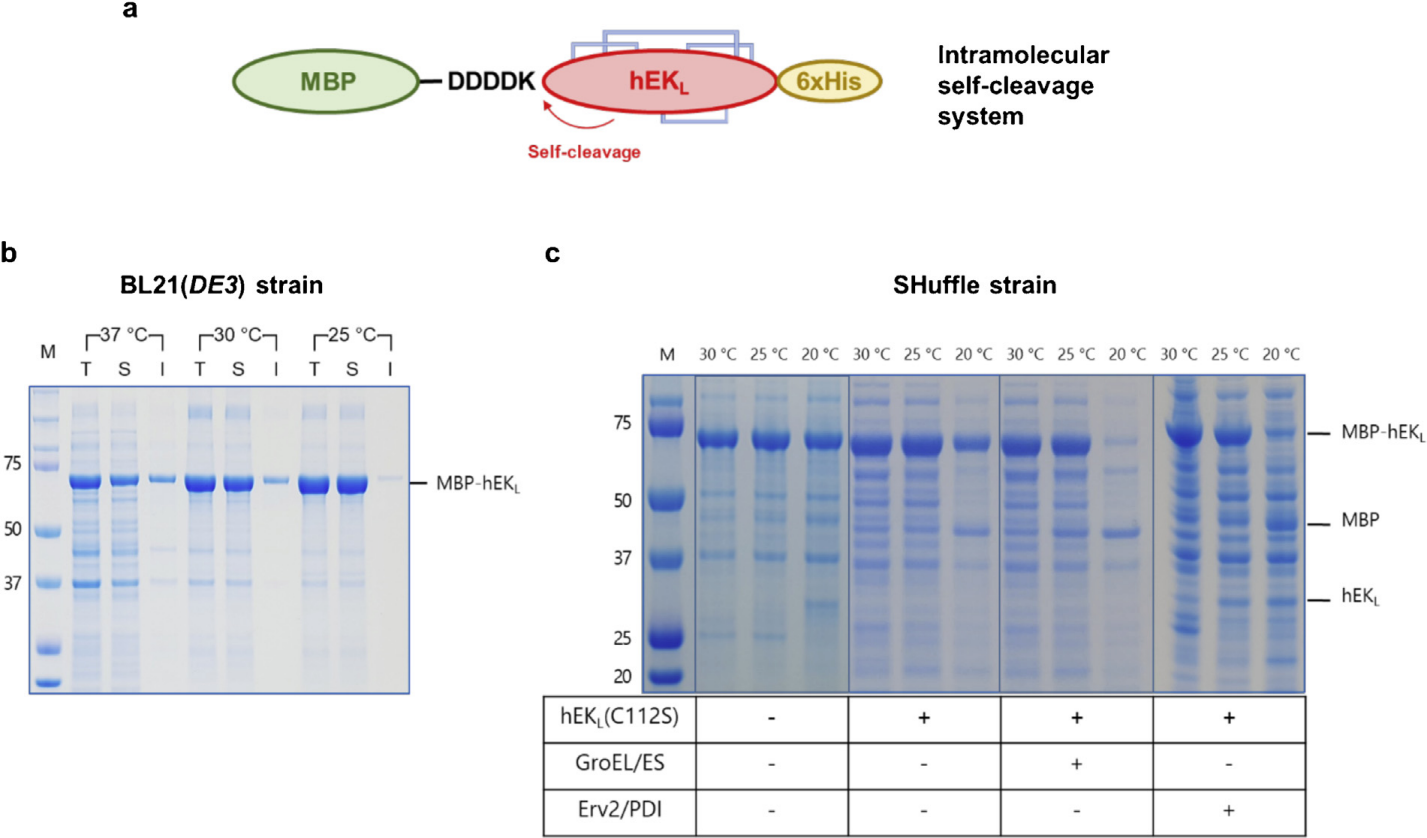
The expression and activity analysis of hEK_L_ in flask culture. (a) Construction of MBP-hEK_L_ fusion connected through the EK cleavage sequence. (b) Expression of MBP-D_4_K-hEK_L_ in *E. coli* BL21 (*DE3*) at different temperatures. (c) The expression of hEK_L_ C112S in *E. coli* SHuffle strain. The blue lane in 1a indicates disulphide bonds. M, Protein marker; I, Insoluble protein; S, soluble protein; T, Total protein.

In this study, we present strategies to produce active hEK_L_ in *E. coli* cytoplasm. We report production of soluble, active hEK_L_ with improved folding efficiency that can be used in-house. To produce active, cytoplasmic hEK_L_ with the correct disulphide bonds, we constructed hEK_L_ fused with MBP through the D_4_K cleavage site and expressed this in *E. coli* cells expressing chaperone proteins (Fig. 1a). A previous report demonstrated expression of soluble and active MBP-tagged hEK_L_ [26]. However, we found that MBP-hEK_L_ was unable to self-cleave, indicating an absence of the enzymatic activity (Figs. S1 and 1b). To test whether removal of MBP could restore the hEK_L_ activity, an hEK_L_ variant was constructed by replacing the D_4_K with the TEV protease recognition site (ENLYFQ). However, hEK_L_ obtained by TEV cleavage of MBP-hEK_L_was still inactive (data not shown). To investigate whether the loss of activity resulted from a limited reduction of disulphide bonds or misfolding, we conducted a refolding process to rearrange disulphide bonds. Detection of self-cleaved forms of refolded hEK_L_ indicated that the refolded enzyme was partially active (Fig. S2). These results demonstrated that MBP fusion enhances the solubility of hEK_L_ but does not allow its correct folding. We speculated that hEK_L_ misfolding might result from incorrect disulphide bonds formed during expression in *E. coli*.

Therefore, to promote the formation of the correct disulphide bonds in *E.coli*-expressed hEK_L_, we employed three strategies: (i) use of a trxB^−^, gor^−^, ahpC*^+^mutant expressing cytoplasmic *DsbC* (SHuffle strain) for oxidative folding, (ii) replacement of the free cysteine with serine (C112S), which bound to heavy chain, to reduce misfolding, and (iii) co-expression of molecular chaperones that isomerize disulphide bonds. First, when the SHuffle strain was used, self-cleaved hEK_L_ was successfully detected, although at a low level (7.9 % of total MBP-D_4_K-hEK_L_), in cells grown at 20 °C (Fig. 1c). Use of the C112S mutated hEK_L_ dramatically improved the ratio of self-cleaved hEK_L_ to up to ∼49.5 % in cells grown at 20 °C, which may be caused by the reduced mispairing of multiple disulphide bonds [12,27]. Remarkably, fully self-cleaved hEK_L_ was detected from cell co-expressing GroEL/ES and Erv2/PDI grown at 20 °C. In particular, the activity was slightly higher upon GroEL/ES co-expression. Notably, hEK_L_ was not visible in the SDS-PAGE gel even when hEK_L_ activity was observed. However, as shown in Fig. S3, when inactivated hEK_L_ was produced by TEVp, hEK_L_ was visible in the SDS-PAGE gel. Therefore, we assumed that the visibility of hEK_L_ in the SDS-PAGE gel was influenced by its folding.

**Fig. 2:**
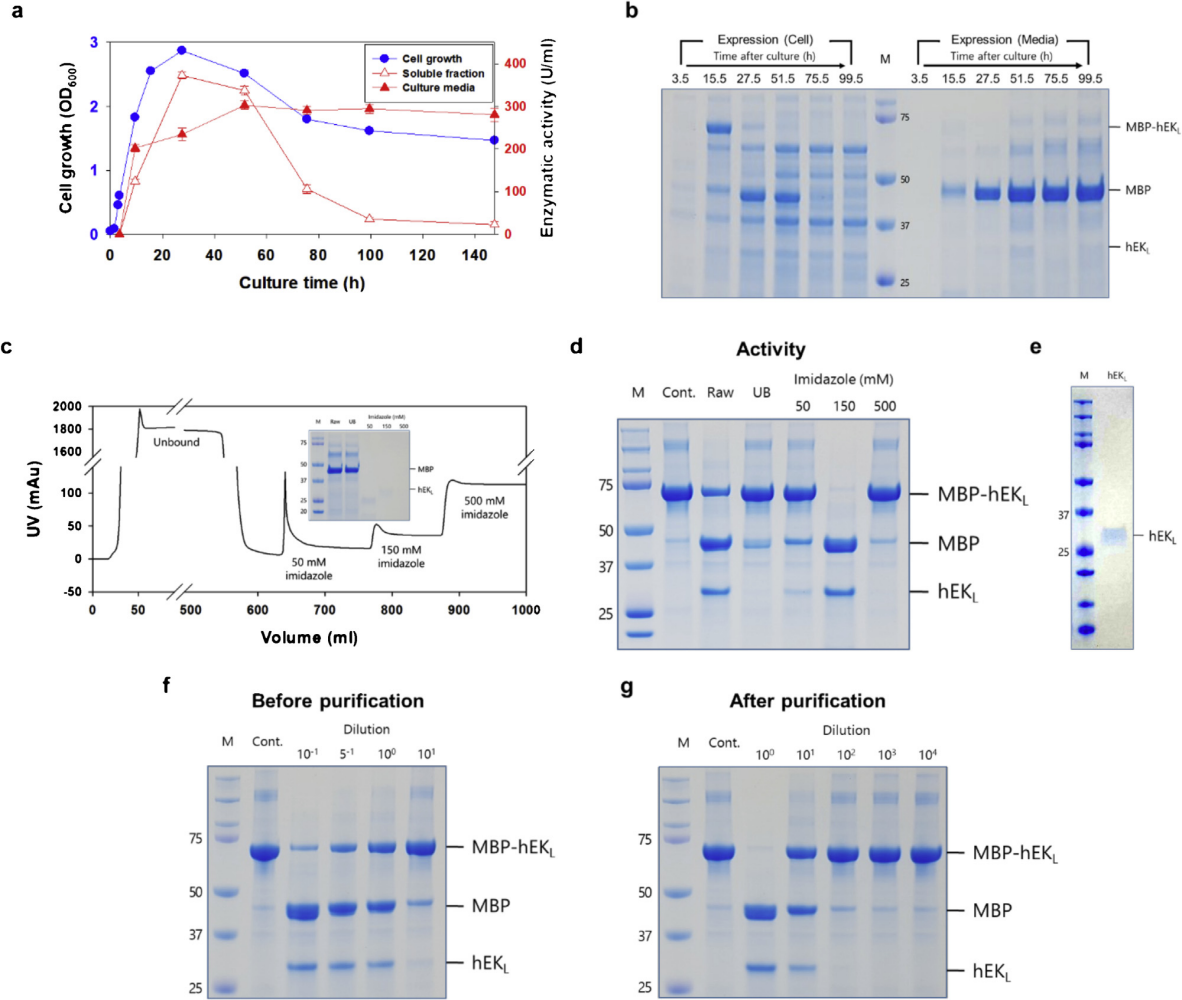
Expression and purification of hEK_L_ C112S. (a) Time-profiles of cell growth and activity of hEK_L_ C112S in flask culture. (b) SDS-PAGE analysis of flask culture samples. (c) Chromatogram of hEK_L_ C112S purification. The inlet indicates SDS-PAGE of each fraction (raw: load fraction, UB: unbounded fraction). (d) Indirect conformation of enzymatic activity of each eluted fraction. MBP-D_4_K-hEK_L_ (25 μg) was treated with 1 μl of purified hEK_L,_ and incubated at 37 °C for 1 h. (e) SDS-PAGE and western blot of purified hEK_L_ C112S. Enzymatic activity of hEK_L_ C112S (f) before purification and (g) after purification. MBP-D_4_K-hEK_L_ (25 μg) was treated with 1 μl of diluted culture supernatant or purified hEK_L,_ and incubated at 37 °C for 1 h. M, Protein marker; Cont., MBP-D_4_K-hEK_L_.

We further monitored the time profiles for cell growth and enzymatic activity of hEK_L_ C112S (Fig. 2a and b). After 27.5 h of culture, the cell growth reached the maximum (2.87 OD_600_) and then sharply decreased. At that time, the hEK_L_ activity in the soluble fraction reached the maximum value (372 U/mL) and then decreased to ∼22 U/mL. In contrast, hEK_L_ in culture supernatants reached the maximum value (303 U/mL) after 75.5 h of culture. These results indicated that hEKL may be released into the extracellular fraction by autolysis of cell.

We attempted to obtain highly pure hEK_L_ C112S from culture supernatants. The culture supernatant of *E. coli* SHuffle expressing pET-30a-MBP-D_4_K-hEK_L_ C112S and pACYC-GroEL/ES was loaded on the affinity chromatography (HisTrap™) along with 1 mM DTT to improve the binding efficacy (Fig. 2c). The enzymatic activity was 306 ± 0 U/mL and 3085 ± 43 U/mL before and after purification, respectively (Fig. 2d–g). A previous report [11] showed that a low-yield hEK_L_ (10 %) can be purified from the culture media of *P. pastoris* using a two-step purification with several pre-treatment steps [11]. However, we could purify hEK_L_ at high purity (>99 %) and yield (>99 %) using a simplified one-step method. Purified hEK_L_ C112S had affinity to GD_4_K-na with *K*_M_ = 0.287 ± 0.079 mM, turnover number *K*_cat_ = 6.725 × 10^4^ ± 1.230 × 10^4^ s^−1^, and catalytic efficiency *K*_M_/*K*_cat_ = 2.385 × 10^5^ mM^−1^ s^−1^.

In conclusion, we could purify soluble and active hEK_L_ at a high yield using an MBP tag, replacing the free cysteine with serine, using *E. coli* strain promoting oxidative folding, co-expressing molecular chaperone that isomerise disulphide bonds, and culturing at low temperature. These findings provide strategies for purification of the complex, multiple disulphide-bonded hEK_L_ from *E. coli*.

## Author contributions

Y.S.K, H. Lee and S.H. Park designed experiments and collected data. Y.K and J.A. supervised the research project and guided the design of experiments. Y.S.K and H.L drafted the manuscript. All authors read the manuscript and agree to submission to Journal of Biotechnology

## Data statement

All data reported in the paper are available from the corresponding author upon reasonable request. Materials and Methods in this study are described in the Supplementary information.

## Declaration of Competing Interest

The authors have no competing interests to declare

## References

[1] Abdelhamid M.A., Motomura K., Ikeda T., Ishida T., Hirota R., Kuroda A. (2014). Affinity purification of recombinant proteins using a novel silica-binding peptide as a fusion tag. Appl. Microbiol. Biotechnol..

[2] Karikari T.K., Turner A., Stass R., Lee L.C., Wilson B., Nagel D.A., Hill E.J., Moffat K.G. (2017). Expression and purification of Tau protein and its frontotemporal dementia variants using a cleavable histidine tag. Protein Expr. Purif..

[3] Carrington J.C., Dougherty W.G. (1988). A viral cleavage site cassette: identification of amino acid sequences required for tobacco etch virus polyprotein processing. Proc. Natl. Acad. Sci. U. S. A..

[4] Gale A.J., Yegneswaran S., Xu X., Pellequer J.L., Griffin J.H. (2007). Characterization of a factor Xa binding site on factor va near the Arg-506 activated protein C cleavage site. J. Biol. Chem..

[5] Waugh D.S. (2011). An overview of enzymatic reagents for the removal of affinity tags. Protein Expr. Purif..

[6] Wang J., Zhang W., Yi Z., Wang S., Li Z. (2012). Identification of a thrombin cleavage site and a short form of ADAMTS-18. Biochem. Biophys. Res. Commun..

[7] Kunitz M. (1939). Formation of trypsin from crystalline trypsinogen by means of enterokinase. J. Gen. Physiol..

[8] Maroux S., Baratti J., Desnuelle P. (1971). Purification and specificity of porcine enterokinase. J. Biol. Chem..

[9] Zheng X.L., Kitamoto Y., Sadler J.E. (2009). Enteropeptidase, a type II transmembrane serine protease. Front. Biosci..

[10] Gasparian M.E., Ostapchenko V.G., Dolgikh D.A., Kirpichnikov M.P. (2006). Biochemical characterization of human enteropeptidase light chain. Biochemistry.

[11] Pepeliaev S., Krahulec J., Černý Z., Jílková J., Tlustá M., Dostálová J. (2011). High level expression of human enteropeptidase light chain in *Pichia pastoris*. J. Biotechnol..

[12] Simeonov P., Berger-Hoffmann R., Hoffmann R., Sträter N., Zuchner T. (2011). Surface supercharged human enteropeptidase light chain shows improved solubility and refolding yield. Protein Eng. Des. Sel..

[13] Liu Y., Ren L., Ge L., Cui Q., Cao X., Hou Y., Bai F., Bai G. (2014). A strategy for fusion expression and preparation of functional glucagon-like peptide-1 (GLP-1) analogue by introducing an enterokinase cleavage site. Biotechnol. Lett..

[14] Gasparian M.E., Ostapchenko V.G., Schulga A.A., Dolgikh D.A., Kirpichnikov M.P. (2003). Expression, purification, and characterization of human enteropeptidase catalytic subunit in *Escherichia coli*. Protein Expr. Purif..

[15] Smith E.T., Johnson D.A. (2013). Human enteropeptidase light chain: bioengineering of recombinants and kinetic investigations of structure and function. Protein Sci..

[16] Collins-Racie L.A., McColgan J.M., Grant K.L., DiBlasio-Smith E.A., McCoy J.M., LaVallie E.R. (1995). Production of recombinant bovine enterokinase catalytic subunit in *Escherichia coli* using the novel secretory fusion partner DsbA. Biotechnology (N Y).

[17] Higaki J.N., Light A. (1986). Independent refolding of domains in the pancreatic serine proteinases. J. Biol. Chem..

[18] Pepeliaev S., Krahulec J., Tlustá M., Černý Z., Jílková J. (2012). Expression and purification of the light chain of human enteropeptidase in *E. coli*. Minerva Biotechnol..

[19] Tengattini S., Rinaldi F., Piubelli L., Kupfer T., Peters B., Bavaro T., Calleri E., Massolini G., Temporini C. (2018). Enterokinase monolithic bioreactor as an efficient tool for biopharmaceuticals preparation: on-line cleavage of fusion proteins and analytical characterization of released products. J. Pharm. Biomed. Anal..

[20] Yi J., Zhang Y.X. (2006). Refolding of the fusion protein of recombinant enterokinase light chain rEKL. Chin. J. Biotechnol..

[21] Skala W., Goettig P., Brandstetter H. (2013). Do-it-yourself histidine-tagged bovine enterokinase: a handy member of the protein engineer’s toolbox. J. Biotechnol..

[22] Tan H., Wang J., Zhao Z.K. (2007). Purification and refolding optimization of recombinant bovine enterokinase light chain overexpressed in *Escherichia coli*. Protein Expr. Purif..

[23] Lemercier G., Bakalara N., Santarelli X. (2003). On-column refolding of an insoluble histidine tag recombinant exopolyphosphatase from Trypanosoma brucei overexpressed in *Escherichia coli*. J. Chromatogr. B.

[24] Liu H., Zhou X., Zhang Y. (2006). A comparative investigation on different refolding strategies of recombinant human tissue-type plasminogen activator derivative. Biotechnol. Lett..

[25] Suh C.W., Park S.H., Park S.G., Lee E.K. (2005). Covalent immobilization and solid-phase refolding of enterokinase for fusion protein cleavage. Process Biochem..

[26] Niu L.X., Li J.Y., Ji X.X., Yang B.S. (2015). Efficient expression and purification of recombinant human enteropeptidase light chain in *Esherichia coli*. Braz. Arch. Biol. Technol..

[27] Ivanenkov V.V., Murphy-Piedmonte D.M., Kirley T.L. (2003). Bacterial expression, characterization, and disulfide bond determination of soluble human NTPDase6 (CD39L2) nucleotidase: implications for structure and function. Biochemistry.

