# Supplementary material for: Expression and purification of soluble and active human enterokinase light chain in Escherichia coli
Source: Biotechnol Rep (Amst). 2021 May 5;30:e00626. doi: 10.1016/j.btre.2021.e00626 (PMC8134707; doi:10.1016/j.btre.2021.e00626)
Supplement: Supplementary file 1 [file mmc1.docx]

**Supplementary information**

**S1. Materials and Methods**

**S1-1. Construction of hEK_L_ expression vectors and strains**

The genes coding for hEK_L_ and hEK_L_ C112S were fused to the EK cleavage site (D_4_K) at its N-terminus, and codon-optimized for *E. coli* (ATUM, Menlo Park, CA, USA). hEK_L_ gene with the TEV protease cleavage site was amplified by PCR using the forward primer:: 5ʹ- GGGATCGAGGGAAGGGAATTCGGAGAAAATCTTTATTTTCAAGGTATTGTTGGCGGCAGC-3ʹ and reverse primer: 5ʹ- GTGGTGGTGGTGGTGCTCGAGTTATTAGTGGTGGTGATG-3ʹ containing *Eco*RI and *Xho*I restriction sites, respectively. The amplified product was ligated into the pre-cut (with *Eco*RI and *Xho*I) (Takara Bio) pET-30a containing MBP, using the In-Fusion Cloning Kit (Takara Bio). The resultant plasmid was designated as pET-30a-MBP-ENLYFQ-hEK_L_. The hEK_L_ and C112S genes with the EK cleavage site were also ligated into pET-30a. Resultant plasmids were designated as pET-30a-MBP-D_4_K-hEK_L_ and pET-30a-MBP-D_4_K-hEK_L_ C112S. The GroEL/ES and Erv2/PDI regions (Chaperone Plasmid Set) (Takara Bio) were amplified by PCR and transformed into the pACYC vector (ATCC® 37033™). The pET-30a-MBP-ENLYFQ-hEK_L_ and pET-30a-MBP-D_4_K-hEK_L_ were transformed into *E. coli* BL21 (DE3) (NEB, Ipswich, MA, USA), and pET-30a-MBP-D_4_K-hEK_L_ C112S was transformed into *E. coli* SHuffle (NEB) using the heat shock method. For the dual vector system, pACYC-GroEL/ES or pACYC-Erv2/PDI with an ampicillin resistance vector were transformed in SHuffle cells with pET-30a-MBP-D_4_K-hEK_L_ C112S.

**S1-2. Expression of hEK_L_ in *E. coli***

hEK_L_ was expressed in *E. coli* using flask cultivation with 200 mL of LB in a 1 L baffled flask. For the seed culture, a single colony was inoculated in 5 mL of LB containing 50 µg/mL kanamycin (50 µg/mL ampicillin was added for dual vectors), and incubated at 37 °C overnight for *E. coli* BL21 (DE3), and 30 °C overnight for *E. coli* SHuffle. For the main culture, 2 mL of seed culture was transferred to 200 mL of LB containing kanamycin (ampicillin was added for dual vectors), and incubated for 25 °C for 24 h, and at 30 °C and 37 °C for 16 h, for *E. coli* BL21 (DE3), 20 °C for 48 h, and at 25 °C and 30 °C for 24 h, for SHuffle. For the production of hEK_L_ C112S, the cells were cultured at 20 °C for 78 h. Recombinant protein expression was induced by the addition of 0.4 mM isopropyl β-D-1-thiogalactopyranoside (IPTG) at an optical density (OD_600_) of 0.6. After IPTG induction, cells were cultured at 20 °C, 25 °C, 30 °C, and 37 °C as described. Cells were harvested by centrifugation (12,000 rpm) at 4 °C for 20 min, and the filtered media was stored (0.45 µm pore size MF Millipore; Merck, Darmstadt, Germany). After washing twice with PBS buffer, the pellet was re-suspended in PBS and lysed by sonication on ice (10% amplitude, pulse: 3 s, on, 5 s, off) for 30 min. Debris was removed by centrifugation at 12000 rpm (4 °C for 20 min) and filtration, using a 0.45 µm filter. Protein expression was analysed using SDS-PAGE by using a 4%–12% Bis-Tris Plus SDS-PAGE gel (Thermo Scientific, Waltham, MA, USA) ran at 170 V (500 mA) for 35 min. The SDS-PAGE gel was stained with InstantBlue (Abcam, Cambridge, UK).

**S1-3. Purification of hEK_L_**

The proteins were purified using the ÄKTAprime plus chromatography system (GE Healthcare, Little Chalfont, UK). For the MBP-hEK_L_ fusion proteins, the soluble fractions of the disrupted *E. coli* BL21 (DE3) cells expressing pET-30a-MBP-ENLYFQ-hEK_L_ or pET-30a-MBP-D_4_K-hEK_L_ were loaded on an MBPTrap HP 5 mL column (GE Healthcare), pre-equilibrated with the binding buffer (50 mM Tris-HCl pH 8, 300 mM NaCl) at 2 mL/min flow-rate. The MBP-hEK_L_ fusion proteins were eluted with 20 mM maltose in binding buffer. hEK_L_ C112S was purified from the culture supernatant of *E. coli* SHuffle expressing pET-30a-MBP-D_4_K-hEK_L_ C112S and pACYC-GroEL/ES. The culture supernatant containing 1 mM DTT was applied to a HisPrep^TM^ FF 16/10 column (GE Healthcare) pre-equilibrated with binding buffer at 2 mL/min flow-rate. After washing column with binding buffer (4 mL/min flow-rate), the impurities were removed using 50 mM imidazole in binding buffer. hEK_L_ C112S was eluted with 150 mM imidazole in binding buffer. The purified hEK_L_ C112S was concentrated into a buffer containing 20 mM Tris-HCl pH 8.0 using the Amicon Ultra-15 Centrifugal Filter 10 kDa cutoff (Merck).

**S1-4. Refolding**

Purified proteins were refolded using a modified method (Pepeliaev et al., 2012): urea (4 M) (Sigma Aldrich, St. Louis, MO, USA) and 1, 4-dithiothreitol (DTT) (1 mM) (Sigma-Aldrich) solutions were added to MBP-D_4_K-hEK_L_. The mixture was incubated at 37 °C for 30 min. The solution was dialyzed against refolding buffer (50 mM Tris-HCl pH 8.5, 1 mM L-glutathione oxidized (GSSG) (Sigma Aldrich), 4 mM L-glutathione reduced (GSH) (Sigma Aldrich), and 20% glycerol) at 4 °C overnight. Refolding was performed by dialyzing against 50 mM Tris-HCl at pH 8.5. After being left overnight at 4 °C, the refolded hEK_L_ was incubated at 37 °C for 2 days to facilitate complete autocatalytic cleavage.

**S1-5. Enzymatic activity of hEK_L_**

The hEKL activity was assessed by the ability of hEK_L_ to cleave the fusion protein’s D_4_K. To test enzymatic activity of the expressed hEK_L_, we defined one unit as 100% cleavage of 25 µg of MBP-D_4_K-hEK_L_ at 37 °C for 1 h. Cleavage reaction was confirmed by SDS-PAGE. To compare activity before and after purification, 10, 2, and 1 µL and 10-fold diluted samples before purification, and 1- to 10^4^-fold serially diluted samples after purification were mixed with 25 µg of MBP-D_4_K-hEK_L_ (37 °C for 1 h). The activity was calculated as the ratio of the cleaved to non-cleaved forms. Activity was quantitated using the cleavage reaction of GD_4_K-β-naphthylamide (GD4K-na) (Sigma Aldrich) at different concentrations. The GD_4_K-na was diluted to final concentrations of 0, 0.1, 0.2, 0.3, 0.4, 0.5, and 0.8 mM in the reaction buffer (25 mM Tris-HCl pH 8.0 with 10 mM CaCl_2_ and 10% DMSO). The reaction was initiated by the addition of hEK_L_ C112S (0.7 nM). Increasing absorbance of the product β-naphthylamine was monitored at 420 nm (excitation wavelength of 337 nm) for 5 min using a SpectraMax® Gemini™ XPS Microplate (Carlsbad, CA, Life Technologies).


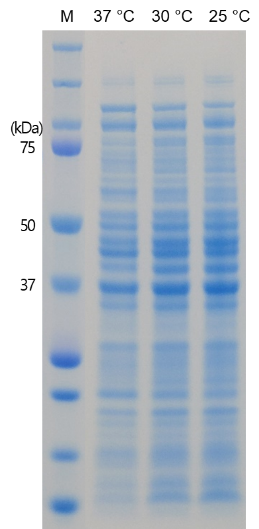


**Figure S1.** The expression profile of wild type *E. coli* BL21 (DE3) (un-induced) at the respective culture temperatures.


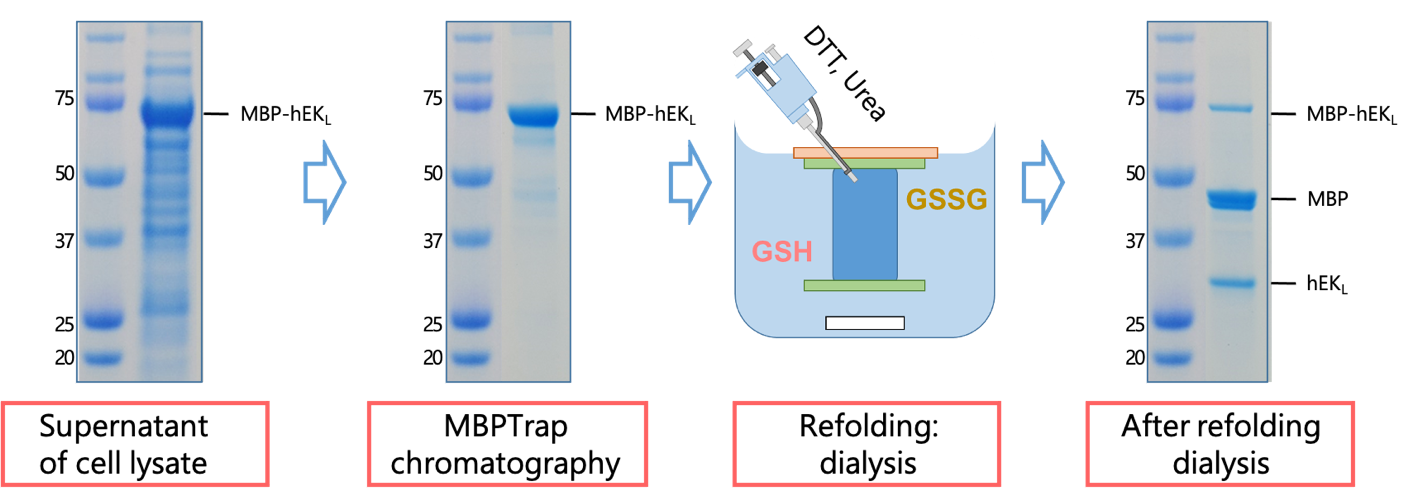


**Figure S2.** The steps and SDS-PAGE images of the refolding process. The MBP-D_4_K-hEKL in the supernatant of cell lysate was purified using MBPTrap chromatography. GSH, Reduced glutathione; GSSG, Oxidized glutathione.


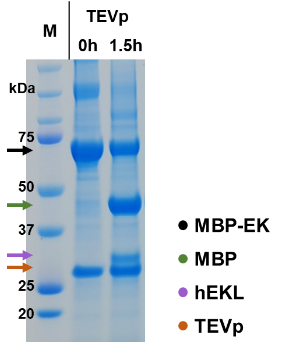


**Figure S3.** SDS-PAGE of hEK_L_ production in the TEV protease cleavage reaction. The fusion protein MBP-ENLYFQ-hEK_L_ (black) was cleaved by TEV protease and separated with MBP and hEK_L_.
